# Contactless Blood Oxygen Saturation Estimation from Facial Videos Using Deep Learning

**DOI:** 10.3390/bioengineering11030251

**Published:** 2024-03-04

**Authors:** Chun-Hong Cheng, Zhikun Yuen, Shutao Chen, Kwan-Long Wong, Jing-Wei Chin, Tsz-Tai Chan, Richard H. Y. So

**Affiliations:** 1 Department of Electrical and Electronic Engineering, Imperial College London, London SW7 2AZ, UK; 2 Department of Computer Science, University of Ottawa, Ottawa, ON K1H 8M5, Canada; zyuen077@uottawa.ca; 3 PanopticAI, Hong Kong Science and Technology Parks, New Territories, Hong Kong, China; shutaochen@panoptic.ai (S.C.); kylewong@panoptic.ai (K.-L.W.); nickchin@panoptic.ai (J.-W.C.); tericchan@panoptic.ai (T.-T.C.); rhyso@ust.hk (R.H.Y.S.); 4 Department of Industrial Engineering and Decision Analytics, The Hong Kong University of Science and Technology, Clear Water Bay, Kowloon, Hong Kong, China

**Keywords:** blood oxygen saturation measurement, deep learning, facial videos, non-contact monitoring, remote health monitoring

## Abstract

Blood oxygen saturation (SpO_2_) is an essential physiological parameter for evaluating a person’s health. While conventional SpO_2_ measurement devices like pulse oximeters require skin contact, advanced computer vision technology can enable remote SpO_2_ monitoring through a regular camera without skin contact. In this paper, we propose novel deep learning models to measure SpO_2_ remotely from facial videos and evaluate them using a public benchmark database, VIPL-HR. We utilize a spatial–temporal representation to encode SpO_2_ information recorded by conventional RGB cameras and directly pass it into selected convolutional neural networks to predict SpO_2_. The best deep learning model achieves 1.274% in mean absolute error and 1.71% in root mean squared error, which exceed the international standard of 4% for an approved pulse oximeter. Our results significantly outperform the conventional analytical Ratio of Ratios model for contactless SpO_2_ measurement. Results of sensitivity analyses of the influence of spatial–temporal representation color spaces, subject scenarios, acquisition devices, and SpO_2_ ranges on the model performance are reported with explainability analyses to provide more insights for this emerging research field.

## 1. Introduction

Human vital signs, such as blood oxygen saturation (SpO_2_), heart rate (HR), respiration rate (RR), blood pressure, and body temperature, are standard parameters used to evaluate a person’s health status [1,2]. Specifically, SpO_2_ readings indicate whether a person has enough oxygen to operate efficiently. SpO_2_ readings are a common metric for trauma management and early detection of diseases like hypoxemia, sleep apnea, and heart diseases [3,4,5].

The COVID-19 pandemic has critically affected many across the globe. According to [6,7], monitoring only an individual’s body temperature is insufficient for detecting COVID-19. Given this limitation, researchers have investigated the feasibility of other vital signs for pandemic control. SpO_2_ is a logical candidate for such monitoring. It has been observed that COVID-infected individuals displayed low SpO_2_ readings before the occurrence of other respiratory symptoms [8,9]. Additionally, some patients have experienced silent hypoxemia, where they exhibit dangerously low SpO_2_ readings without signs of respiratory distress [10]. Wide deployment of an accurate tool that can conveniently and rapidly monitor SpO_2_ would greatly enhance a global ability to control inflammatory infectious diseases such as COVID-19.

Currently, SpO_2_ is generally measured non-invasively using pulse oximeters and other wearable devices [11,12]. However, contact-based devices have usability limitations and are impractical for long-term monitoring. Usage for extended periods can cause discomfort and are unsuitable for those with skin sensitivity [13]. Moreover, using contact-based devices for health monitoring may facilitate the spread of infectious diseases. Therefore, contactless approaches for SpO_2_ measurement have emerged as highly desirable.

Over the last decade, several contactless SpO_2_ measurement approaches have been proposed. Researchers have used a variety of cameras, from infrared cameras [14] and high-quality monochrome cameras equipped with special filters [15,16,17,18] to off-the-shelf webcams [19,20,21,22,23], to estimate SpO_2_ by capturing subtle light intensity changes on the face. Deep learning techniques have achieved state-of-the-art performance for the remote measurement of physiological signs such as HR [24,25,26,27,28,29,30,31,32,33,34,35,36,37,38,39] and RR [36,39,40,41,42,43,44,45,46,47]. However, remote SpO_2_ measurement is still in its infancy, with only a few papers using convolutional neural networks (CNNs) to predict SpO_2_ from RGB facial videos [48,49,50]. Additionally, most existing methods are evaluated on private self-collected datasets, preventing a fair comparison of algorithmic performance [51].

In this paper, we utilize a spatial–temporal representation—that is, a spatial–temporal map (STMap), as proposed in [52]—to encode SpO_2_-related physiological information from videos recorded by several consumer-grade RGB cameras. Each STMap is fed into various 2D CNNs for predicting SpO_2_ in an end-to-end manner. In addition, We explore the explainability of the model and visualize feature maps of each hidden layer to uncover the process of how it addresses input data. This illustrates the advantage of using an STMap instead of taking the spatial average as input. Moreover, we make use of a public benchmark dataset, VIPL HR [52,53], to conduct our experiments and analysis. This research investigates the feasibility of utilizing a spatial–temporal map for remote SpO_2_ measurement and evaluates the proposed method on a public dataset for fair comparison. Our deep learning approach offers these contributions to ongoing research:

- It is trained and evaluated on a large-scale multi-modal public benchmark dataset of facial videos.
- It outperforms conventional contactless SpO_2_ measurement approaches, showing potential for applications in real-world scenarios.
- It provides a deep learning baseline for contactless SpO_2_ measurement. With this baseline, future research can be benchmarked fairly, facilitating progress in this important emerging field.

## 2. Literature Review

### 2.1. Contact-Based SpO_2_ Measurement

Today, pulse oximeters are being widely utilized to monitor SpO_2_ in a non-invasive manner. The principle underlying SpO_2_ measurement through pulse oximetry is known as the Ratio of Ratios [54,55]. Pulse oximeters contain Light Emitter Diodes (LEDs) that generate two different light wavelengths, 660 nm (red) and 940 nm (infrared), to measure the different absorption coefficients of oxygenated hemoglobin (HbO2) and deoxygenated hemoglobin (Hb) [56]. The photodetector inside the pulse oximeter analyzes the light absorption of these two wavelengths and produces an absorption ratio from which the SpO_2_, as a percentage, can be determined from the table in [57]. Healthy SpO_2_ values generally range from 95% to 100% [58]. Equation (1) illustrates how pulse oximeters measure SpO_2_.

(1) $$SpO_{2}=\frac{C_{HbO_{2}}}{C_{Hb}+C_{HbO_{2}}}\times 100\%$$

where C_HbO2_ is the concentration of HbO_2_ and C_Hb_ is the concentration of Hb.

### 2.2. SpO_2_ Measurement with RGB Cameras

Since smartphones have become ubiquitous in our daily lives, researchers have explored the possibility of SpO_2_ measurements through a smartphone camera [11,12]. Using these methods, subjects place their fingertips on top of the smartphone camera, and SpO_2_ is estimated based on the reflected light captured by the camera. However, since most smartphone cameras are visible imaging sensors—that is, they only capture light in the visible portion of the spectrum—they cannot capture infrared wavelengths. To overcome this deficiency, Scully et al. [11] proposed to replace the infrared component of the Ratio of Ratios principle with the blue wavelength, since the difference between the absorption coefficients of HbO2 and Hb are very similar at the two wavelengths [12,59,60,61]. Equation (2) illustrates the Ratio of Ratios principle for SpO_2_ with an RGB camera.

(2) $$SpO_{2}=A-B\frac{\left(AC_{RED}\right)/\left(DC_{RED}\right)}{\left(AC_{BLUE}\right)/\left(DC_{BLUE}\right)}$$

where AC_BLUE_ and AC_RED_ represent the standard deviations of the blue and red color channels, respectively while DC_BLUE_ and DC_RED_ represent the mean of the blue and red color channels, respectively. A and B are experimentally evaluated coefficients that are determined by identifying the line of best fit between the ratios of the red and blue channels and the SpO_2_ estimated by a ground truth device. Following Equation (2), remote SpO_2_ measurement with an RGB camera was further validated in [21,22,23,48,50]. However, only two methods used deep learning and were tested on a public benchmark dataset [48,49].

### 2.3. Deep Learning-Based Remote Vital Sign Monitoring

Over the last decade, many deep learning-based methods have been developed for remote vital sign monitoring, with many studies focusing on HR [24,25,26,27,28,29,30,31,32,33,34,35,36,37,38,39], followed by RR [36,39,40,41,42,43,44,45,46,47]. In general, the underlying principle behind these methods is remote photoplethysmography (rPPG). When body tissues are illuminated by surrounding light, tiny fluctuations in reflected light intensities due to variation in the concentration of hemoglobin can be captured by conventional cameras, producing the so-called rPPG signal [62,63]. After extracting the rPPG signal, subsequent vital signs such as HR or RR can be obtained by further signal processing.

Among the deep learning-based methods for remote SpO_2_ measurement based on RGB facial videos [48,49,50], Hu et al. [48] utilised a multi-model fusion approach and took advantage of the Ratio of Ratios principle. Hamoud et al. [49] used an XGBoost Regressor [64] to measure SpO_2_ with the features extracted by a pre-trained CNN. Akamatus et al. [50] made use of spatial–temporal input that is based on the AC and DC components of the Ratio of Ratios principle.

### 2.4. Spatial–Temporal Representation for Vital Sign Estimation

For remote physiological measurement from facial videos, the crucial information is extracted from the changes in pixel intensity of the subject’s face. Since contactless methods are inherently susceptible to noise such as illumination changes and head movements [24], a spatial-averaging operation is generally performed on the region of interest (face) to enhance the quality of the extracted signal. Niu et al. [52] proposed an rPPG-based spatial-temporal representation, spatial–temporal map (STMap), that is widely used for HR estimation as well as face anti-spoofing [39,52,65,66,67,68]. The STMap, a low-dimensional spatial–temporal representation in which physiological information of the original video is embedded, can be directly fed into a CNN, which learns and develops a function for mapping a connection between the STMap and the output vital sign. To the best of our knowledge, there is no existing work that has applied rPPG-based STMaps to predict SpO_2_. Given the success of spatial–temporal representations for estimating HR, this motivates us to utilize a similar approach for remote SpO_2_ measurement.

## 3. Materials and Methods

### 3.1. Spatial–Temporal Map Generation

**Figure 1:**
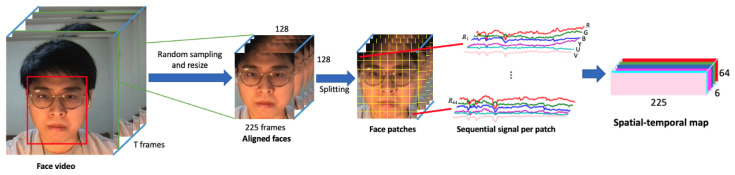
Process of generating a spatial–temporal map in RGB + YUV color spaces.

As shown in Figure 1, we followed an approach similar to that proposed in [52] to generate spatial–temporal maps (STMaps). For each video, we randomly sampled 225 consecutive frames and used a face detector (OpenFace [69]) to obtain the subject’s face location. The facial frames were down-sampled to 128 × 128 using an average pooling filter (kernel size = 16 and stride = 16) to reduce noise and image dimension. Each frame was then split into 64 patches (8 × 8, from $R_{1}$ to $R_{64}$), and average pooling was applied to each patch for noise removal. Let *P*(*x*,*y*,*t*,*c*) be the intensity value of the pixel with the coordinate(*x*,*y*) of the *t*th frame of the video at *c* color space, and the average pooling of these patches can be denoted as

(3) $$V_{c,i}\left(t\right)=\frac{\sum_{x,y\in R_{i}}P\left(x,y,t,c\right)}{A_{R_{i}}}$$

where $A_{R_{i}}$ represents the area of the patch $R_{i}$. Then, for each patch, we have a sequential signal with length of 225 for each color space c, which is $V_{c,i}$ = $\left\{V_{c,i}\left(1\right),V_{c,i}\left(2\right),\ldots ,V_{c,i}\left(225\right)\right\}$. For the case of combining RGB and YUV color space, the value of c should be 6. Lastly, these sequential signals are concatenated to form an STMap, a 2D map generated from a video with embedded SpO_2_-related information.

Other than the traditional RGB color space, an STMap can also be generated from different or a combination of multiple color spaces [65]. In this paper, we transformed the RGB color space to YUV and YCrCb color spaces through Equations (4) and (5), respectively:

(4) $$\begin{array}{c} \begin{array}{c} Y=0.299\times R+0.587\times G+0.114\times B\\ U=-0.169\times R-0.331\times G+0.5\times B+128\\ V=0.5\times R-0.149\times G-0.081\times B+128 \end{array} \end{array}$$

(5) $$\begin{array}{c} \begin{array}{c} Y=0.299\times R+0.587\times G+0.114\times B\\ Cr=(R-Y)\times 0.713+128\\ Cb=(B-Y)\times 0.564+128 \end{array} \end{array}$$

**Figure 2:**
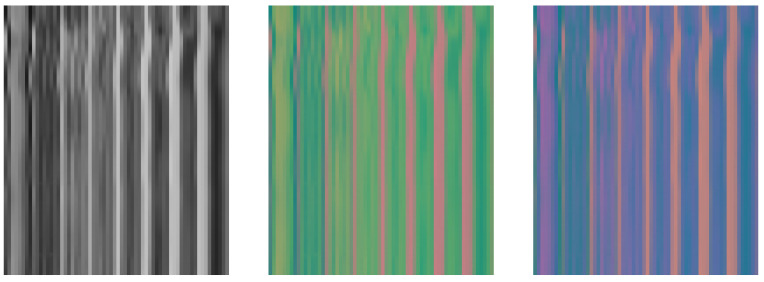
Examples of the spatial–temporal maps (STMaps) in RGB (**left**), YUV (**middle**), and YCrCb (**right**) color spaces generated from the VIPL-HR dataset.

The *c* color dimensions for each face patch were concatenated to produce the final spatial–temporal representation of size 225 × 64 × *c*. Figure 2 shows a visual example of the STMaps generated from the different color spaces.

### 3.2. SpO_2_ Estimation Using CNNs

We framed SpO_2_ estimation as a regression problem and utilized 2D CNNs to predict a single SpO_2_ value from an STMap. The STMaps were resized to 225 × 225 to match the input size of the CNNs. We selected and compared three state-of-the-art CNN architectures that are commonly utilized in computer vision tasks, namely ResNet-50 [70], DenseNet-121 [71], and EfficientNet-B3 [72], which were pre-trained with the ImageNet [73] dataset. The last layer of each model was replaced with a regression layer. Table 1 shows their model complexities.

### 3.3. Dataset

We trained and tested our models on STMaps generated from the VIPL-HR. The VIPL-HR dataset (https://vipl.ict.ac.cn/resources/databases/201811/t20181129_32716.html) (accessed on 20 June 2023) dataset [52,53], is a public-domain dataset originally proposed for remote HR estimation. Since SpO_2_ readings were also recorded during the data collection, VIPL-HR can also be used for bench-marking contactless SpO_2_ measurement methods. The dataset contains 2378 RGB and 752 near-infrared (NIR) facial videos of 107 subjects (79 males and 28 females, mostly Asians) recorded by four acquisition devices (web camera, smartphone frontal camera, RGB-D camera, and NIR camera). The length of each video is around 30 s, with a frame rate of around 30 frames per second.

**Figure 3:**
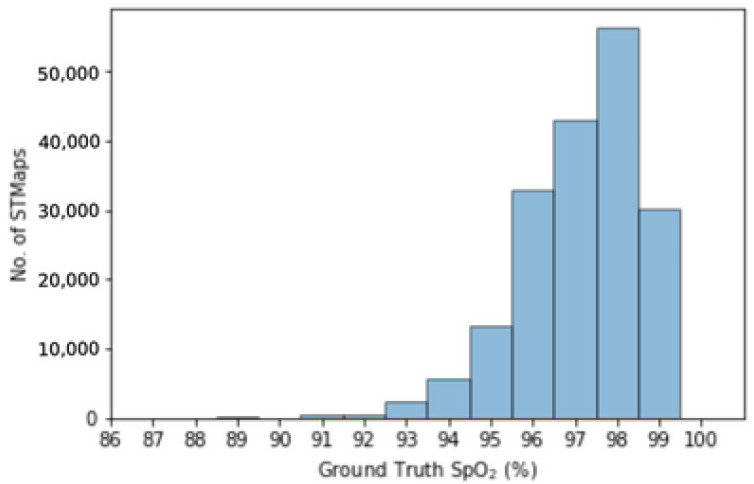
Ground truth SpO_2_ (%) distribution of STMaps generated from the VIPL-HR dataset.

For our experiments, we utilized RGB videos of subjects sitting naturally in nine scenarios as follows: (1) at 1 m, (2) while performing large head movements, (3) while reading a text aloud, (4) in a dark environment, (5) in a bright environment, (6) at a long distance (1.5 m instead of 1 m), (7) after doing exercise for 2 min, (8) while holding the smartphone, and (9) while holding the smartphone and performing large head movements. Specific details of the data collection process are listed in [53]. The large variety in the scenarios contributes to the generalizability of the proposed method for different applications. Figure 3 illustrates the distribution of ground truth SpO_2_ values for STMaps generated from the VIPL-HR dataset.

### 3.4. Evaluation Metrics

We utilized the following performance metrics to evaluate the performance of SpO_2_ prediction:

- Mean absolute error (MAE) = $\frac{\sum_{i=1}^{N}\left|x_{i}-y_{i}\right|}{N}$;
- Root mean square error (RMSE) = $\sqrt{\frac{\sum_{i=1}^{N}{\left(x_{i}-y_{i}\right)}^{2}}{N}}$.

where xi is the predicted SpO_2_ and yi is the ground truth SpO_2_ in unit of percentage (%).

### 3.5. Training Settings

To ensure fair evaluation, we performed five-fold subject cross-validation, during which we first separated the subjects into small bins according to the distribution of the SpO_2_ values of each subject. Each small bin contained at least 5 subjects. Within each bin, the subjects were randomly split into 5 groups. This process guaranteed that the SpO_2_ values of each fold were equitably distributed. We conducted a Friedman chi-squared test among different folds and the *p* value was recorded as 0.273, which meant we could not refuse the H_0_ hypothesis that the samples were drawn from the same distribution. The final MAE and RMSE results were obtained by averaging over the five folds.

For the training data, we randomly sampled 225 consecutive frames 70 times for each video in the training set to generate STMaps. There are at least 113,068 STMaps for training in each fold. For model training, we used the AdamW optimizer [74] and batch size of 32 on a NVIDIA RTX 3080 GPU. The initial learning rate was set to 0.0001 with a weight decay of 0.001. The RMSE loss function was also utilized for all models. It takes around 12 h to train a single model.

### 3.6. Feature Map Visualization

**Figure 4:**
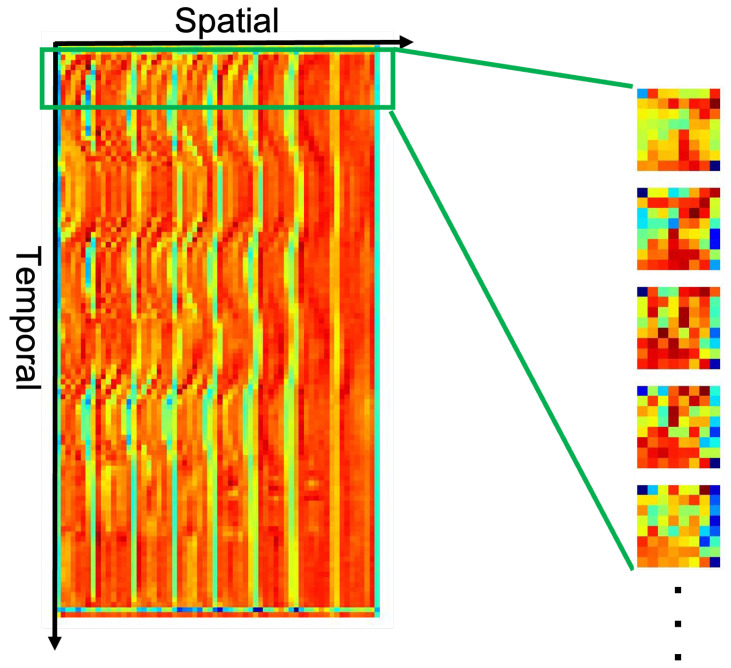
Example of a recorded feature map from the first hidden convolutional layer in blocks. During forward propagation, different color channels were fused; therefore, we average the feature maps over different channels within the layer. Each column corresponds to a patch along the temporal axis and each row corresponds to one frame. For visualization, each row was transformed back to an 8 × 8 square sequence. The subject’s face can still be recognized from the reshaped squares.

While deep learning-based approaches have shown remarkable performance in different vital sign estimation tasks, it is of great interest to uncover what the neural network has learned. A video stream from the dataset was presented to the network and forward-propagated to predict SpO_2_, during which the responses of hidden convolutional layers on different levels were recorded. The extracted feature maps were averaged over all channels within each layer. As the response map of each layer was a 2D STMap, each row of the feature map that corresponded to a timestamp was detached and separately transformed back to an 8 × 8 image for better visualization. This process is illustrated in Figure 4.

We applied this process to all convolutional layers to transform the 2D STMap back to interpretable 2D squared image sequences. The results are shown in the next section.

## 4. Results and Discussion

### 4.1. Performance on STMaps Generated from Different Color Spaces

As mentioned in [52,75], during the generation of the spatial–temporal representation, selecting an appropriate color space can reduce head motion artifacts and improve the overall signal quality of STMaps. To investigate the impact of color space on SpO_2_ estimation, we compared the performance of STMaps generated from RGB, YUV, concatenated RGB and YUV, and YCrCb color spaces.

Among the trained models, EfficientNet-B3 trained on concatenated YCrCb STMaps (EfficientNet-B3 + YCrCb) achieved the lowest MAE and RMSE (Table 2) but the combination of YCrCb color space with the other two models resulted in unsatisfactory performance. Moreover, all deep learning models achieved a relatively satisfactory performance when trained on RGB STMaps. This indicates that the introduction of additional color spaces during STMap generation will not improve the deep learning model’s performance for SpO_2_ estimation, but the selection of appropriate color space will affect the performance. Specifically, RGB color space seems to achieve the most stable performance.

### 4.2. Performance on Different Subject Scenarios and Acquisition Devices

As EfficientNet-B3 + RGB achieved a relatively stable and good performance in the previous experiment, we used EfficientNet-B3 + RGB as our deep learning benchmark for subsequent analysis. We evaluated the performance of our deep learning method against the conventional Ratio of Ratios algorithm for contactless SpO_2_ estimation (Equation (2)) with coefficients A and B from previous works [21,22]. We further investigated the performance of these methods in different subject scenarios and acquisition devices in the VIPL-HR dataset. We also included the performance of other deep learning methods [48,49] that have been tested on the VIPL-HR dataset. Additionally, the deep learning method proposed by Hu et al. [48] was first trained on another public dataset, PURE [76], and then fine-tuned on VIPL-HR.

Table 3 highlights that all deep learning methods significantly outperform the conventional Ratio of Ratios algorithm on the VIPL-HR dataset by at least 30% [21] with an up to 66.7% [22] reduction in RMSE. Moreover, the results are within the error range (4%) according to the international standard for a pulse oximeter that can be used for clinical purposes [77], showing the capability of deep learning-based approaches for real-world applications. Notwithstanding, due to the variance in the model’s performance between subjects, the historical trends of SpO_2_ measurements are often a better indication of the subject’s health status than a single measurement at one point in time.

**Figure 5:**
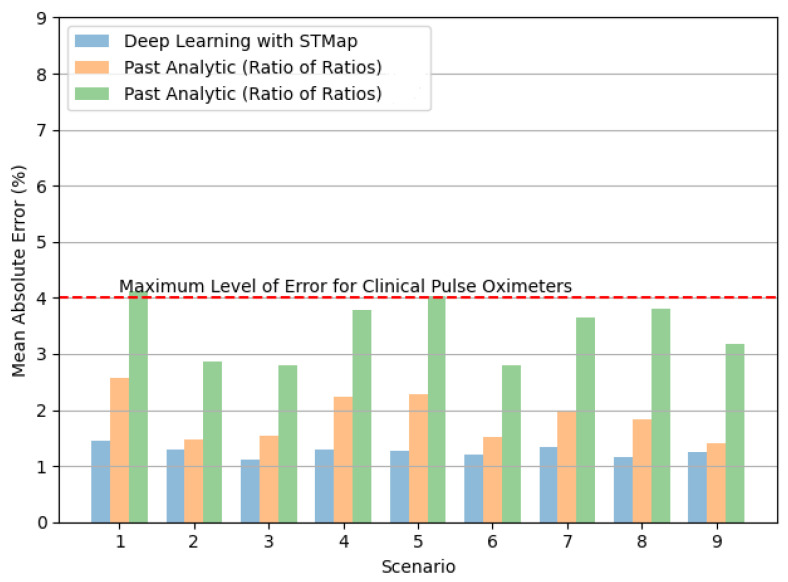
Comparison of mean absolute error (MAE) in remote SpO_2_ estimation by deep learning with STMap and past analytic methods (Green refers to [22], Orange refers to [21]) for different subject scenarios of the VIPL-HR dataset.

**Figure 6:**
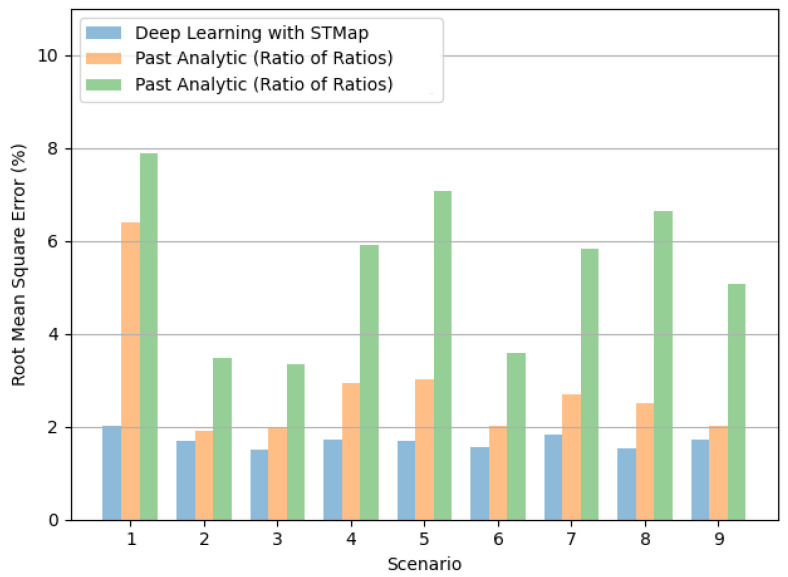
Comparison of root mean square error (RMSE) in remote SpO_2_ estimation by deep learning with STMap and past analytic methods (Green refers to [22], Orange refers to [21]) for different subject scenarios of the VIPL-HR dataset.

Figure 5 and Figure 6 show the performance of the tested methods in different subject scenarios in the VIPL-HR dataset (Section 3.3). The deep learning method consistently achieved the lowest MAE (Figure 5) and RMSE (Figure 6) in all cases. Moreover, it is worth noting the significant performance difference between methods in Scenarios 4 and 5, indicating the deep learning method’s potential to address illumination variations.

**Figure 7:**
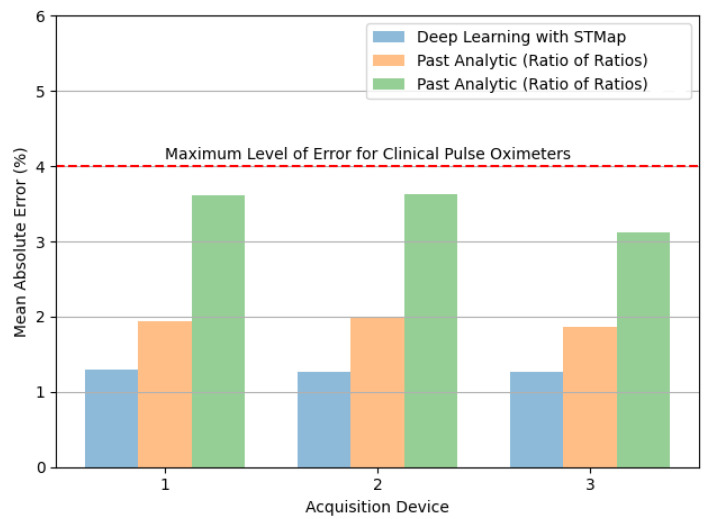
Comparison of mean absolute error (MAE) in remote SpO_2_ estimation by deep learning with STMap and past analytic methods (Green refers to [22], Orange refers to [21]) for different acquisition devices (1 = Web Camera, 2 = Smartphone Frontal Camera, 3 = RGB-D Camera) of the VIPL-HR dataset.

**Figure 8:**
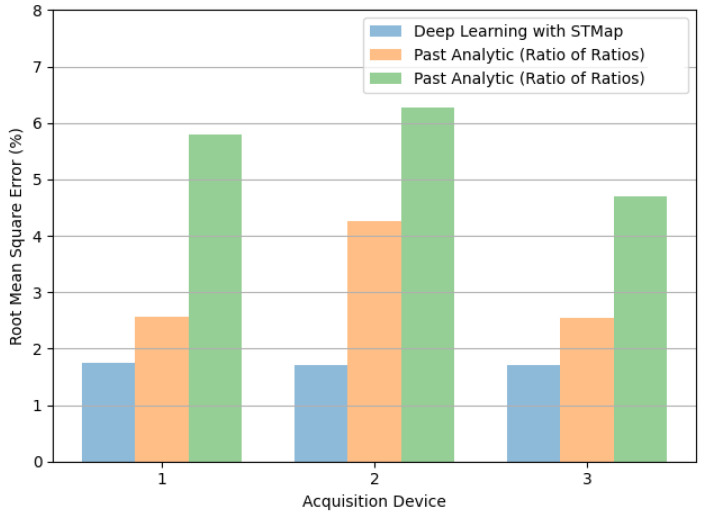
Comparison of root mean square error (RMSE) in remote SpO_2_ estimation by deep learning with STMap and past analytic methods (Green refers to [22], Orange refers to [21]) for different acquisition devices (1 = Web Camera, 2 = Smartphone Frontal Camera, 3 = RGB-D Camera) of the VIPL-HR dataset.

Figure 7 and Figure 8 illustrate the performance of the tested methods on different acquisition devices, including: (1) Logitech C310 web camera (960 × 720, 25fps), (2) HUAWEI P9 frontal camera (1920 × 1080, 30fps), and (3) RealSense F200 RGB-D camera (1920 × 1080, 30fps) in the VIPL-HR dataset. Consistent with the results of subjects in different scenarios, the deep learning method achieved the lowest MAE (Figure 7) and RMSE (Figure 8) for all acquisition devices.

### 4.3. Performance over Different SpO_2_ Ranges

Inspired by Li et al. [78], we analyzed the performance of remote SpO_2_ estimation methods over different SpO_2_ ranges. The SpO_2_ value of a healthy person is usually between 95% and 100% [58]. Based on this classification, we separated the data into two groups: normal (SpO_2_ ≥ 95%) and abnormal (SpO_2_ < 95%).

From Table 4, we observe that the deep learning method outperforms the Ratio of Ratios algorithm in both normal and abnormal SpO_2_ ranges. However, the model’s MAE and RMSE in the normal range (0.978 and 1.288, respectively) are significantly lower than those in the abnormal range (3.077 and 3.563, respectively). The model’s increase in prediction error in the abnormal range may be because the distribution of the training dataset contains fewer low SpO_2_ values. Similar to the conclusion drawn in [78] for predicting HR values in the higher and lower ranges, the challenge of predicting abnormal SpO_2_ measurements should be a focus of future works.

### 4.4. Feature Maps Learned by CNN Model

**Figure 9:**
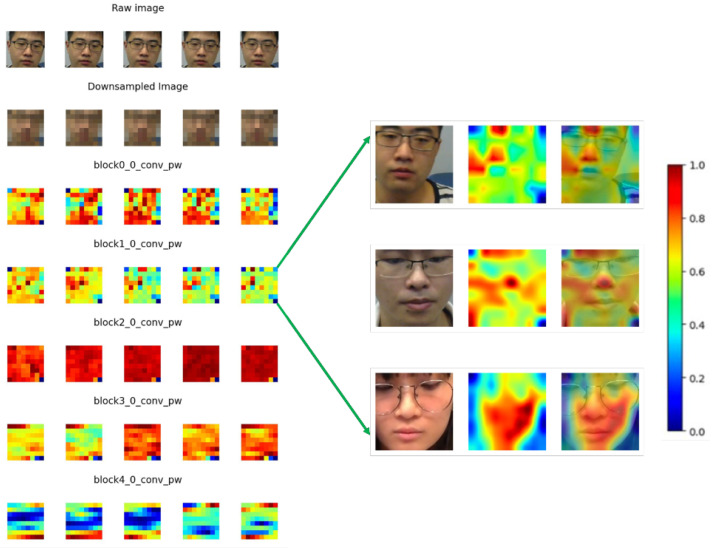
Visualization of feature maps. The left column illustrates the feature maps of hidden convolutional layers for the given input video stream after training for SpO_2_ prediction. The first 5 convolutional layers were selected from the sequential blocks of Efficientnet-b3 model. The right column illustrates the raw image, overlayed with the interpolated feature maps extracted from hidden layer block1_0_conv_pw for 3 subjects in the VIPL-HR dataset.

In Figure 9, the raw input frame and its down-sampled image are shown on the top two rows and the responses of different hidden layers in the Efficientnet-b3 model are shown sequentially. Here, only the first five convolutional layers are displayed as the feature maps of higher-level convolutional layers are hard to recognize. The untrained model is shown on the left as a sub-figure for comparison while the results for the trained model are shown on the right side. All the values were normalized between 0 and 1.

It can be seen from the feature maps on the left side of Figure 9 that, in the initial block0_0_conv_pw layer, the outline of the subject is still recognizable by the human eye. For the block1_0_conv_pw layer, some regions are emphasized with larger weights and others are less stressed. To find the physical meaning of these regions, we aligned the feature map with the raw input frame by applying bicubic interpolation to retain the same resolution as input raw images and overlayed them; the results of this process are displayed on the right side of Figure 9.

After interpolation, it can be seen clearly from the right side of Figure 9 that, in the block1_0_conv_pw layer, different face parts were assigned different weights. More specifically, the forehead, nose, and cheeks were assigned a larger weighting while other regions such as the torso or spaces without the human face carried less weight. This result is consistent with findings from many rPPG-related studies, where the forehead, left and right cheeks are often selected as the regions of interest (ROIs) as they carry more physiological information [21,22].

For the hidden convolutional layers in higher levels of the model, the patterns are illegible and therefore not discussed in our study.

## 5. Conclusions and Future Research Direction

In this paper, we proposed and evaluated a new deep learning method for remote SpO_2_ measurement from facial videos in the VIPL-HR public database. We encoded the facial videos into STMaps, low-dimensional spatial–temporal representations containing physiological information of the subject, and directly used them as model inputs for training and testing. Our results indicate that the proposed deep learning method outperforms the conventional Ratio of Ratios technique by reducing the RMSE up to 66.7% when compared across different subject scenarios, acquisition devices, and SpO_2_ ranges. This sets a new bench-marking baseline for upcoming research. The visualization of feature maps demonstrated that ROIs around the forehead, nose, and cheeks carry more weight for SpO_2_ estimation. These findings increase the explainability of the models.

Regarding the direction of future research, we posit that improving the face detection process can generate more representative STMaps and enhance the model’s robustness, especially for videos of subjects with large head movements. We expect that a face detector that operates on a per-frame basis, while taking into consideration the dimensional requirements to generate the STMap, can optimize the signal-to-noise ratio of the spatial–temporal representations. Furthermore, as demonstrated by Niu et al. [65], region-of-interest selection can be incorporated to capture areas that may contain stronger physiological signals. Additionally, further investigation could be directed toward assessing the impact of resizing the STMaps to match the CNN’s input dimensions, as this procedure may introduce additional noise to the model. Other feature maps with hidden layers could be investigated to elucidate the mechanism of SpO_2_ prediction. Moreover, most of the subjects that participated in the VIPL-HR dataset are Asians with Fitzpatrick Scale skin type III and IV [79]. Therefore, the proposed method may be biased to people with these skin types and may not perform considerably on darker skin tones (type VI), which is a common concern in remote vital sign monitoring [80,81,82]. Finally, we would like to collect more data of subjects with different skin tones and abnormal SpO_2_ readings or to simulate low SpO_2_ values through an approach similar to the one used in [59]. Additional data coverage of subjects with diverse skin tones and abnormal SpO_2_ values can contribute to the development of more robust and accurate models for contactless SpO_2_ measurement.

## Figures and Tables

**Table 1:** Number of parameters (Params) and floating point operations per second (FLOPs) of the selected CNN architectures.

Model	Params	FLOPs
EfficientNet-B3 [72]	9.2 M	1.0 B
ResNet-50 [70]	26 M	4.1 B
DenseNet-121 [71]	8 M	5.7 B

**Table 2:** Performance of selected deep learning models trained on STMaps generated from different color spaces for SpO_2_ estimation.

	RGB		YUV		RGB + YUV		YCrCb	
Model	MAE	RMSE	MAE	RMSE	MAE	RMSE	MAE	RMSE
	(%)	(%)	(%)	(%)	(%)	(%)	(%)	(%)
EfficientNet-B3 [72]	1.274	1.710	1.304	1.756	1.279	1.707	1.273	1.680
ResNet-50 [70]	1.309	1.741	1.307	1.750	1.321	1.781	1.423	1.939
DenseNet-121 [71]	1.284	1.722	1.357	1.783	1.296	1.713	1.421	1.860

**Table 3:** Performance of deep learning methods and past analytic methods (Ratio of Ratios) for SpO_2_ estimation.

Method	MAE (%)	RMSE (%)
Deep Learning with STMap (EfficientNet-B3 + RGB)	1.274	1.710
Deep Learning [48]	1.000	1.430
Deep Learning [49]	1.170	-
Past Analytic (Ratio of Ratios) [22]	3.334	5.137
Past Analytic (Ratio of Ratios) [21]	1.838	2.489

**Table 4:** Performance of deep learning with STMap (EfficientNet-B3 + RGB) and past analytic methods (Ratio of Ratios) for SpO_2_ estimation in normal (≥95%) and abnormal (<95%) ranges.

	Normal		Abnormal	
Method	MAE	RMSE	MAE	RMSE
	(%)	(%)	(%)	(%)
Deep Learning with STMap (EfficientNet-B3 + RGB)	0.978	1.288	3.077	3.563
Past Analytic (Ratio of Ratios) [22]	3.140	4.972	6.798	7.496
Past Analytic (Ratio of Ratios) [21]	1.690	2.264	4.482	5.034

## Data Availability

Publicly available dataset was analyzed in this study. This dataset can be found here: https://vipl.ict.ac.cn/resources/databases/201811/t20181129_32716.html, accessed on 26 February 2024.
